# Impact of COVID-19 on the mental health of optometry students at a higher education institution: A case study

**DOI:** 10.4102/aveh.v80i1.652

**Published:** 2021-09-21

**Authors:** Yusuf Simjee, Zothile Mncwabe, Keihara Sindhrajh, Rabia Khan, Maseeha Seedat, Letiwe Xulu, Sibahle Zondi, Nishanee Rampersad

**Affiliations:** 1 Discipline of Optometry, School of Health Sciences, University of KwaZulu-Natal, Durban, South Africa

**Keywords:** mental health, COVID-19, student wellness, higher education institution, Depression Anxiety Stress Scale questionnaire

## Abstract

**Background:**

The coronavirus disease 2019 (COVID-19) outbreak poses serious threats to the physical and mental health of individuals worldwide. The lockdown strategy and social distancing regulations adopted in South Africa have disrupted the day-to-day life activities of all people including students.

**Aim:**

To explore the impact of COVID-19 on the mental health of optometry students at a higher education institution.

**Setting:**

The study population included optometry students currently registered at the University of KwaZulu-Natal, in Durban, South Africa.

**Methods:**

The study adopted a case study research design and used an online questionnaire and follow-up interviews to collect data. The Depression Anxiety Stress Scale (DASS-21) questionnaire that assesses depression, anxiety and stress subscales was used. Data were analysed using descriptive and inferential statistics. The interview data were analysed using thematic content analysis.

**Results:**

A total of 147 participants responded to the online questionnaire and consisted of majority black people (*n* = 98), female (*n* = 114) and second year (*n* = 58) students. The majority of the participants had normal scores for depression (*n* = 97), anxiety (*n* = 79) and stress (*n* = 107). Only a few participants had scores indicating severe or extremely severe depression (*n* = 13), anxiety (*n* = 23) and stress (*n* = 5). Follow-up interviews were conducted with 10 participants and of these, seven reported that COVID-19 had a negative impact on their ability to concentrate and they felt anxious about examining patients. Factors such as stigma and judgment were perceived as hindrances to seeking mental health help.

**Conclusion:**

Most participants had normal scores for depression, anxiety and stress; however, some participants presented with abnormal scores. The impact of the COVID-19 pandemic on mental health should be highlighted and higher education authorities should plan and provide appropriate services to improve the quality of life of affected students.

## Introduction

Coronaviruses are a large family of viruses that may cause respiratory infections. The most recent coronavirus disease, which is the coronavirus disease 2019 (COVID-19), is an infectious disease caused by severe acute respiratory syndrome coronavirus 2 (SARS-CoV-2).^1,2^ The outbreak of COVID-19 was identified in Wuhan, China in December 2019.^2^ The World Health Organization (WHO) declared the COVID-19 outbreak as a public health emergency of international concern on 30 January 2020 and recognised it as a pandemic on 11 March 2020. Apart from the crucial challenges to the well-being of humans, COVID-19 also poses a serious threat to the mental health of individuals.^3,4,5,6^ In addition to fear of death from COVID-19, death amongst family members and friends and the resulting impact on mental health are important aspects that need to be recognised and addressed. As of 06 September 2021, there were 221.9 million cases of COVID-19 worldwide with just over 1.5 million deaths.^7^ In South Africa, as of 06 September 2021, there were almost 2.8 million confirmed cases with 83 617 deaths^8^ and these figures reflect a serious scenario with respect to the rapid spread of infection. Psychological stress and other mental health illnesses have been highlighted as having a marked connection with COVID-19.^9^ This is because high levels of stress and anxiety are natural responses towards any unnatural situation.^10^

According to the WHO, mental health is:

[*A*] state of well-being in which an individual realises his or her own abilities, can cope with the normal stresses of life, can work productively and is able to make a contribution to his or her community.^11^

Governments in various countries around the world have taken precautionary measures because of COVID-19 and imposed travel and trade restrictions that have caused life changes for people around the world. In South Africa, people were asked to maintain social distancing, wash their hands for at least 20 seconds, wear cloth face coverings in public and stay at home except when obtaining essential items like medical care and groceries. Preventive measures such as social distancing and self-isolation during this pandemic are likely to negatively affect mental health as face to face interaction is critical to well-being.^12^ Furthermore, face to face interaction is known to decrease depression^13^ and therefore long periods of limited social interaction may have a negative impact on this aspect. Whilst social media helps to stay connected to one another, it does not replace the need for human contact.^12^ Brooks et al.^14^ suggested that individuals in quarantine may experience psychological distress in the form of anxiety, anger confusion and post-traumatic stress symptoms. According to a survey conducted by the South African Depression and Anxiety Group (SADAG) in May 2020, 65% of the total sample (*n* = 1214) felt stressed or very stressed during the lockdown period in South Africa.^15^ In their study, challenges reported by the SADAG included anxiety and panic, financial stress and pressure, depression, poor family relations, feelings of suicide and substance abuse.^15^ In another study, conducted in July 2020 involving adults in the United States by the Kaiser Family Foundation, 53% of participants reported that worry and stress of COVID-19 had a negative impact on their mental health.^16^ Participants also reported difficulty with sleeping or eating, increases in alcohol consumption or substance use and worsening chronic conditions.^16^

As a result of the precautionary measures and imposed restrictions of the COVID-19 pandemic, the education system has been affected.^17,18^ Different countries have introduced and implemented various educational policies, ranging from complete closure in Germany and Italy to targeted closure in England and Scotland.^19,20^ Additionally, over 100 countries worldwide have imposed nationwide closure of educational facilities at all levels from basic to higher education.^19^ In this way, the COVID-19 pandemic has created significant challenges for the global education community.^18,21^ The pandemic quickly resulted in closure of universities and colleges around the world so that social distancing and self-isolation practices could help to flatten the infection curve and reduce fatalities from the disease.^22^ Consequently, the COVID-19 pandemic brought about a global shift to remote learning. This transition greatly impacted the education system by replacing the traditional contact teaching and learning practices with online teaching and learning practices.^18,22^ Online learning protocols are currently being employed globally by educational institutions and this includes a rapid change from face-to-face classes to online learning systems.^22^ As a result, the closure of educational institutions along with the increasing rate of COVID-19 infections has resulted in higher levels of anxiety, uncertainty and stress amongst the university students and community.^23,24^ The aim of this study was to assess the impact of COVID-19 on the mental health, in terms of depression, anxiety and stress, of optometry students registered at a higher education institution.

## Methodology

A case study research design was used to investigate the impact of COVID-19 on the mental health of optometry students. The study population included optometry students currently registered at the University of KwaZulu-Natal (UKZN) Westville campus in Durban. The study included participants from all races and both genders, aged 18 years and older and registered optometry students at the UKZN, Westville campus. Participants were excluded if they were 17 years or younger or not registered optometry students at the UKZN Westville campus. At the time of the study, there were 241 optometry students registered at the UKZN Westville campus. A saturated sample consisting of all registered undergraduate optometry students at UKZN were invited to participate in the study through a link sent via a social media platform (WhatsApp).

Data for the study were collected via an online questionnaire and a follow-up online interview. An online questionnaire was considered as a safe and efficient method of data collection during the global COVID-19 pandemic. The online anonymous questionnaire was administered over a 7-week period (03 August 2020 to 20 September 2020). Each participant anonymously answered the online questionnaire that was created on Google Forms. The online questionnaire comprised three sections and took approximately 10 to 15 minutes to complete. The first section contained the study aim, objectives, information document and agreement to participate in the study. The second section contained questions related to demographics and participant information and the third section contained the questions in the Depression Anxiety Stress Scale (DASS-21) questionnaire that was used to assess depression, anxiety and stress as has been used in other studies.^9,25,26,27,28^ The interviews were conducted on Zoom and took approximately 10 to 15 min to complete. In the online questionnaire, participants were asked if they were interested in participating in a follow-up interview. After the online questionnaire was closed, participants who responded positively to a follow-up interview were contacted and if they were still willing, the interview was set for a mutually convenient date and time. The online follow-up interview, conducted via Zoom, was recorded and saved on a password protected computer. All participants voluntarily read and acknowledged the online information document and consent form before completing the questionnaire and gave verbal consent before being interviewed.

There are two versions of the DASS questionnaire, a full 42-item version and an abbreviated 21-item version. Both versions of the DASS questionnaire serve to detect the core symptoms of the three negative emotional states of depression, anxiety and stress.^29^ The short version of the DASS questionnaire was used in the present study wherein there are seven items in each of the three subscales (depression, anxiety and stress).^25,29^ In the DASS-21 questionnaire, the depression scale assesses dysphoria, hopelessness, devaluation of life, self-deprecation, the lack of interest or involvement, anhedonia and inertia and is represented by items 3, 5, 10, 13, 16, 17 and 21.^25,29^ In the DASS-21 questionnaire, the anxiety scale assesses autonomic arousal, skeletal muscle effects, situational anxiety and the subjective experience of anxious affect and is represented by items 2, 4, 7, 9, 15, 19 and 20.^25,29^ In the DASS-21 questionnaire, the stress scale is sensitive to levels of chronic non-specific arousal and assesses difficulty relaxing, nervous arousal and being easily upset or agitated, irritable or over-reactive and impatient and is represented by items 1, 6, 8, 11, 12, 14 and 18.^25, 29^ Participants were asked to use a four-point rating scale to rate the extent to which they had experienced each state over the COVID-19 lockdown thus far (0 - did not apply to me at all, 1 - applied to me some of the time, 2 - applied to me a good part of the time, 3 - applied to me most of the time). The scores for depression, anxiety and stress in the DASS-21 questionnaire were determined by summing the scores for the relevant items as per the scoring instructions in the DASS manual.^29^

The DASS-21 questionnaire is reliable, has strong internal consistency and has been translated into many languages, thus making it usable in various cultural groups.^9,29^ It has adequate psychometric properties and has been widely used in many studies to assess symptoms of psychological distress amongst both clinical and non-clinical groups.^9,30,31,32,33,34^ The anxiety subscale differentiates between people with panic disorder and those with other anxiety or depressive disorders; the depression subscale differentiates between people with depressive disorders and those with anxiety disorders and the stress subscale differentiates between people with generalised anxiety disorders and those with phobic or obsessive-compulsive disorders. The internal consistency, divergent validity and convergent validity have shown to be culturally indifferent,^27,35,36^ given that almost all 21 items in the questionnaire are culturally free, thus allowing it to be used amongst different ethnic groups.^26^ The DASS-21 questionnaire also has Cronbach alpha values of 0.81, 0.89 and 0.78 for the depression, anxiety and stress subscales, respectively, which indicates good internal consistency and reliability.^25^

Semi-structured interviews were conducted and an interview guide with predetermined questions and probes was used to guide the interview and achieve optimum use of the interview time.^37^ A sampling frame of all participants who were willing to participate in a follow-up interview was created. Participants were selected for the follow-up interview using simple random sampling and of the 16 participants who were selected, six were excluded as they did not respond to the invitation or indicated that they were no longer available. Thus, the follow-up interviews were conducted on 10 participants who volunteered to participate after completing the online questionnaire, were available and selected using simple random sampling. The interviews were conducted and recorded on Zoom and common themes from each interview were extracted. The interview guide contained open-ended questions to allow for more in-depth interview data to be produced.^37^ The interviews were recorded and data were captured verbatim to allow for more accurate recording of responses.^38^

Data from the online questionnaire were analysed with the Statistical Package for Social Sciences (SPSS) version 27 using descriptive and inferential statistics. Data are presented using frequencies, means, standard deviations and percentages. The independent sample *t*-test was used to compare depression, anxiety and stress scores between males and females. A *p*-value of less than 0.05 was considered statistically significant. The interview data were analysed using thematic content analysis which involved identification of common themes, ideas and patterns in the interview transcripts.^37,39^

### Ethical considerations

Data collection occurred after ethical approval was obtained from the Humanities and Social Sciences Research Ethics Committee (HSSREC/00001350/2020) at the UKZN.

## Results

### Characteristics of the sample

A total of 147 participants responded to the online questionnaire comprising 33 males (22.4%) and 114 females (77.6%). In terms of the level or year of study, 16 (10.9%) participants were in first year, 58 (39.5%) participants were in second year, 33 (22.4%) participants were in third year and 40 participants (27.2%) were fourth year students. The sample consisted of majority black people (*n* = 98, 66.7%) followed by Indian people (*n* = 43, 29.3%), Caucasian people (*n* = 4, 2.7%) and mixed race people (*n* = 2, 1.4%). There were 51 (34.7%) participants who lived in rural areas, 71 (48.3%) participants who lived in urban areas and 25 (17.0%) participants who lived in semi-urban areas. There were 112 (76.2%) participants who lived with their parents whilst 35 (23.8%) participants did not live with their parents. Information about COVID-19 was obtained mostly from the Internet (*n* = 79, 53.7%) and television (*n* = 52, 35.4%) and a few participants used other sources including applications (*n* = 9, 6.1%), radio (*n* = 6, 4.1%) and newspapers (*n* = 1, 0.7%). There were 121 (82.3%) participants who were afraid to get COVID-19 whilst 26 (17.7%) participants were not afraid. A total of 15 (10.2%) participants had family members who had contracted the COVID-19 virus at the time of data collection.

### Depression Anxiety Stress Scale-21 questionnaire scores

Table 1 shows the frequency of responses per question in the DASS-21 questionnaire for the 147 participants. Most of the participants responded ‘did not apply to me at all’ for all questions with the exception of three questions (numbers 5, 6 and 9). In these three questions, which probed depression, stress and anxiety respectively, the majority of participants reported that they experienced these symptoms some of the time (Table 1).

**TABLE 1:** Frequency of responses for the questions in the DASS-21 questionnaire.

Number	Question	Did not apply to me at all	Applied to me some of the time	Applied to me a good part of the time	Applied to me most of the time
1(s)	I find it hard to wind down.	92	43	8	4
2(a)	I am aware of dryness of my mouth.	95	34	11	7
3(d)	I cannot experience any positive feeling at all.	99	34	12	2
4(a)	I experienced breathing difficulty (e.g. excessively rapid breathing, breathlessness in the absence of physical exertion).	113	30	4	-
5(d)	I found it difficult to work up the initiative to do things.	47	62	24	14
6(s)	I tend to over-react to situations.	44	63	29	11
7(a)	I experienced trembling (e.g. in the hands).	111	29	5	2
8(s)	I felt that I was using a lot of nervous energy.	72	51	18	6
9(a)	I am worried about situations in which I might panic and make a fool of myself.	48	52	34	13
10(d)	I feel that I had nothing to look forward to.	94	31	17	5
11(s)	I find myself getting agitated.	71	48	25	3
12(s)	I find it difficult to relax.	64	54	20	9
13(d)	I feel downhearted and blue.	81	45	18	3
14(s)	I am intolerant of anything that kept me from getting on with what I was doing.	63	56	21	7
15(a)	I feel I was close to panic.	80	45	20	2
16(d)	I am unable to become enthusiastic about anything.	85	33	21	8
17(d)	I feel I am not worth much as a person.	103	26	13	5
18(s)	I feel that I was rather touchy.	94	38	13	2
19(a)	I am aware of the action of my heart in the absence of physical exertion (e.g. sense of heart rate increase, heart missing a beat).	89	32	18	8
20(a)	I feel scared without any good reason.	92	35	13	7
21(d)	I feel that life was meaningless.	115	19	7	6

s, stress; a, anxiety; d, depression.

**FIGURE 1:**
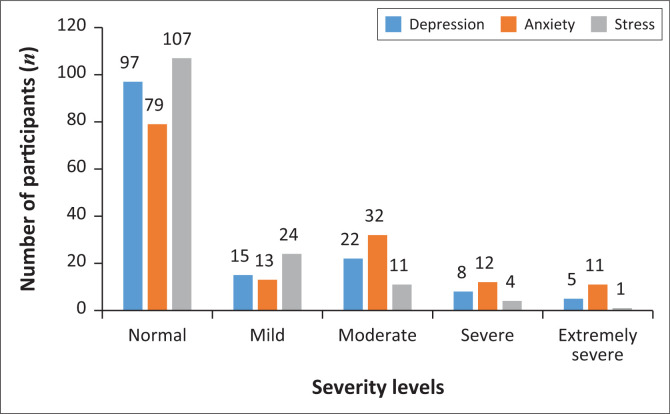
Severity levels for depression, anxiety and stress subscales in participants (*n* = 147) as measured using the Depression Anxiety Stress Scale-21 questionnaire.

The different levels of severity for the three subscales in the DASS-21 questionnaire are shown (Figure 1). Overall, 97 participants had normal scores for depression whilst only 13 had experienced severe or extremely severe depression. Similarly, 79 participants had a normal anxiety score whilst only 11 participants were found to be severely anxious in response to the pandemic (Figure 1). Most of our participants (*n* = 107) had a normal score for the stress subscale. However, five participants were classified with severe or extremely severe symptoms of stress.

Table 2 shows the mean scores for the three subscales stratified for gender and level or year of study. Male participants had higher mean scores for depression, anxiety and stress (Table 2). The mean values for depression, anxiety and stress in males were 10.67, 8.61 and 10.79, respectively, with a total score of 30.06 (Table 2). The mean values for depression, anxiety and stress for females were 7.49, 7.75 and 9.98, respectively, with a total score of 25.23 (Table 2). Despite the gender difference in mean scores for depression, anxiety and stress, these differences were not significant (*p* ≥ 0.08). In terms of level or year of study, fourth year students had the highest scores for depression, anxiety and stress as well as the total score (Table 2).

**TABLE 2:** Depression Anxiety Stress Scale-21 scores (means and standard deviations) stratified for gender and level of study.

DASS-21 subscale	Gender		Level or year of study			
	Males (*n* = 33)	Females (*n* = 114)	First (*n* = 16)	Second (*n* = 58)	Third (*n* = 33)	Fourth (*n* = 40)
Depression score	10.67 ± 9.04	7.49 ± 8.07	9.50 ± 10.13	8.00 ± 8.61	6.18 ± 7.22	9.65 ± 8.08
Anxiety score	8.61 ± 7.20	7.75 ± 6.80	7.75 ± 6.85	8.21 ± 6.92	6.30 ± 6.60	9.00 ± 7.08
Stress score	10.79 ± 7.16	9.98 ± 7.74	10.25 ± 9.49	9.72 ± 7.62	8.36 ± 6.05	12.25 ± 7.68
**Total score**	**30.06 ± 20.86**	**25.23 ± 20.32**	**27.50 ± 23.03**	**25.93 ± 21.45**	**20.85 ± 17.36**	**30.90 ± 19.90**

### Interview results

The interviews were conducted with 10 participants of which eight were female and two were male. Of the 10 participants, six were Indian people, three were black people and one was a white person. There were five participants in the fourth year, three in the second year and two in the third year. The interviews were conducted during level 1 of the COVID-19 lockdown in South Africa. At the time of data collection, study participants in third and fourth year had returned to UKZN in July 2020 and were examining patients at external clinics and public hospital sites. Study participants who were in first and second year had not returned to UKZN since March 2020. The themes that emerged from the interviews are presented below.

#### Theme 1: Impact of COVID-19 on completion of degree, studying and family responsibilities

Participants described the impact of COVID-19 on the completion of their degree as stressful, demotivating to study and hard to concentrate. This was indicated by one of the participants:

‘I was feeling very demotivated, it was very difficult to get back on board to studying because I had no idea what was going to happen the rest of the year’. (Participant 2, fourth-year student, 22 September 2020)

Another participant mentioned: ‘So far everything is going smoothly’ (Participant 3, second-year student, 25 September 2020). They felt that the pace of learning had slowed down as opposed to what it used to be as a result of the use of online learning. Some were concerned about losing time because of COVID-19 and not making enough hours to complete their degree of study. This was indicated by one of the participants:

‘I feel like we are going to have difficulties completing or getting our patient numbers and hours. Even though the Health Professionals Council of South Africa has decreased our patient numbers that we need, I feel like COVID-19 has affected us in that way’. (Participant 9, fourth-year student, 25 September 2020)

Not completing that the degree would have an effect on their family responsibilities as it alters the plans of the family, for example, parents retiring later or parents emigrating. One participant mentioned: ‘My family is waiting for me to graduate so I can help them as well’ (Participant 6, fourth-year student, 24 September 2020). One participant was concerned: ‘Our future employers won’t have that much faith in us’ (Participant 9, fourth-year student, 25 September 2020). Participants indicated that it would affect them mentally as they had a goal of finishing their optometry degree of study within 4 years. One participant reported: ‘I feel like I am tired, I need to finish’ (Participant 9, fourth-year student, 25 September 2020). Another said: I would not want to repeat the year’ (Participant 5, second-year student, 25 September 2020).

#### Theme 2: Concerns over academic delays, education and academic performance

A participant was concerned: ‘That I won’t make enough patient numbers and I have to redo the year because of that, which is out of my control’ (Participant 6, fourth-year student, 24 September 2020). Online learning took time to adjust, data availability and network connection were also important issues. For example, a participant mentioned: ‘The environment that I was in was not suitable for me to study and I had laptop issues’ (Participant 8, second-year student, 26 September 2020). Participants (*n* = 9) felt that online learning was effective and understood that most lecturers tried their best to support and provide effective learning; however, some still felt that the lockdown had affected learning and caused an academic delay. A participant mentioned: ‘Online learning was effective in its own way, but I also had to be effective’ (Participant 4, fourth-year student, 25 September 2020). Participants (*n* = 7) reported that it was difficult to have the self-discipline needed for online learning. Another felt that they needed more pressure to complete tasks quicker and avoid procrastination. A participant reported: ‘It taught me a lot of self-discipline and to be able to time my things’ (Participant 9, fourth-year student, 25 September 2020). A participant indicated: ‘Even though the results might be good in numbers, I think people have lost a lot in terms of gaining knowledge’ (Participant 2, fourth-year student, 22 September 2020). They feel that they learn best through face-to-face conversations and that the COVID-19 lockdown had affected their practical skills. A participant mentioned: ‘Having that direct conversation with the lecturer helps them (students) learn better’ (Participant 2, fourth-year student, 22 September 2020). Some participants (*n* = 5) were concerned over the practical aspects and were afraid that once they returned to UKZN, they would be rushed into learning many techniques that required much time and observation.

#### Theme 3: Consulting with patients after returning from the COVID-19 lockdown

Participants described examining patients as ‘scary’. As there are many ways of contracting COVID-19 and being in close contact with patients, especially when performing techniques like ophthalmoscopy and slit lamp, made participants scared and self-conscious about aspects such as touching their own face without sanitising. Participants (*n* = 7) reported being anxious about patient behaviour, for instance, a participant reported: ‘That if a patient were to cough, it would make them question “did this patient have COVID-19?”’ (Participant 4, fourth-year student, 25 September 2020). Many participants felt that they would have to change their routine during an examination and be more cautious than they would have been previously. They felt that wearing masks and face shields were very uncomfortable, overwhelming and inconvenient during an examination for both the patient and examiner. Some participants would often read the lips of soft-spoken patients and the use of the face mask prevented this from happening. Those who did not return to university said that they would feel very anxious and nervous to consult with patients. Many participants (*n* = 7) were concerned about being in contact with patients because they were aware of the high risk of contracting and spreading COVID-19. For example, a participant said:

‘We may not suspect them but then again patients could be asymptomatic and that was stressful, thinking I would bring home a disease or something because my parents are immunocompromised’. (Participant 7, fourth-year student, 27 September 2020)

Another participant mentioned: ‘It was a risk, it was a lot of anxiety, anxiousness, not knowing’ (Participant 10, fourth-year student, 24 September 2020). In contrast, two participants reported that they were excited to examine patients again. For example, one of these participants indicated: ‘To be honest I was excited but also, I was nervous’ (Participant 9, fourth-year student, 25 September 2020). The other participant reported: ‘I’d be quite excited, it would be a good thing, I’d feel happy it’s finally over, finally I can see patients and not panic or stress’ (Participant 6, fourth-year student, 24 September 2020).

#### Theme 4: Worries because of COVID-19 in terms of accommodation, finances and relationships

COVID-19 has caused a strain on participants financially as some family members lost jobs, those who were self-funded were required to pay extra for private accommodation to protect against COVID-19 and even travelling to UKZN had become difficult. Many participants (*n* = 7) anticipated starting to earn and help their families. It was mentioned that some participants tried to avoid their family members even when wearing proper protection in fear of transmitting COVID-19. It was mentioned: ‘The only worry that I have is visiting family because some people can be asymptomatic, so I don’t like being around my older members of family at all even if I have my mask on’ (Participant 5, second-year student, 25 September 2020). Some participants (*n* = 4) reported that being away from their friends had been difficult for them as they felt as if they did not have anyone to talk to. This was indicated by a participant: ‘That distance between you and your friend because of the rules of COVID-19, it does affect [*you*] because you just become stressed and you don’t have anyone to talk to’ (Participant 2, fourth-year student, 22 September 2020). Others felt anxious at the thought of reuniting with friends. Some reported that they preferred face-to-face interaction to communicate with peers and did not like using electronic devices. In terms of partner relationships, COVID-19 played a role in ending some relationships and this influenced the state of mind of the participants which in turn affected their studying. For participants who were living at home, accommodation and family were not significantly affected. This was indicated by one participant: ‘With family relationships it didn’t really affect me that much because I live with my family and accommodation was not affected’ (Participant 4, fourth-year student, 25 September 2020).

#### Theme 5: COVID-19 on daily living and overall health

Participants described being indoors all the time as ‘difficult’, ‘depressing’ and ‘frustrating’ and when they did go outdoors, they felt that having to wear a mask as ‘suffocating’. Many (*n* = 5) said that they missed the freedom pre-COVID-19. Some claimed to be more forgetful and reported: ‘I feel like my mental capacity has shrunken somehow and now I easily forget stuff’ (Participant 2, fourth-year student, 22 September 2020). Some mentioned they were not physically active during the lockdown and because of the lack of social interaction and family that they felt alone. A few participants found that their physical health improved. One participant mentioned:

‘With being at home I’ve had time to set my own routine to things, I’ve had time to eat healthier and cook healthier meals so that was a big up but then there is the down of not wanting to be near family and worrying about every little cough or sneeze. Definitely stress was a big contender.’ (Participant 5, second-year student, 25 September 2020)

Other participants felt that even though the lockdown had given them more time, it took away their routine. One participant mentioned: ‘When you don’t have any structure and any routine then it does make you sink into depression, so I think just keeping busy has helped’ (Participant 7, fourth-year student, 27 September 2020). A participant described the lockdown as: ‘An unexpected holiday which wasn’t a holiday because it was a stressful holiday’ (Participant 2, fourth-year student, 22 September 2020). A participant reported: ‘There was not much to do I got bored and then I realised time was going and a lot of time had passed and then I started getting anxious, feeling worried about the semester’ (participant 6, fourth-year student, 24 September 2020).

#### Theme 6: The impact and extent of COVID-19 on mental health

When asked if COVID-19 has affected your mental health, most participants responded yes. Many participants (*n* = 6) indicated that they experienced stress, depression, anxiety, panic attacks, sleeping and eating problems because of COVID-19. One participant mentioned that drug or alcohol and self-harm, or harm of others was not a big factor and when drug or alcohol was used, it was to help decrease their stress levels. Participants acknowledged that mental health was an important aspect during the lockdown. A participant reported: ‘The more time I spent at home, the more I realise I need to better my mental health’ (Participant 3, second-year student, 25 September 2020). Some participants (*n* = 5) mentioned that stress was the only factor that affected their mental health and this mostly related to completion of their degree of study. One participant said: ‘It was just stress related to university mainly’ (Participant 5, second-year student, 25 September 2020). Some of the aspects related to completing their degree of study included making enough patient numbers and hours, online learning, thinking of the practical aspect and skills that were being missed out.

#### Theme 7: The need for mental health help and the type of help

Many participants (*n* = 9) felt that their mental health was under control and that they could manage it on their own and found comfort in having the support of friends and family. A participant indicated: ‘I don’t think I’m at the stage where I would need counselling or anything because I have a good support system from my family and friends’ (Participant 7, fourth-year student, 27 September 2020). As the lockdown restrictions are easing, participants felt that their mental health would be better. Others mentioned that if they needed help, they would seek it. Some did not feel that they could trust anybody to talk about their mental health. Many participants reported that advice on finances, time management and appearing for assessments and writing examinations would be of benefit to them. Participants (*n* = 4) believed that having the optometry department, friends and family would be useful as sometimes when they feel stuck in a situation, someone from the outside could provide a simple solution. A participant had suggested:

‘It would be a lot less stressful if the university communicated with us more frequently and gave us more information as to what was happening because that would reduce a lot of anxiety’. (Participant 6, fourth-year student, 24 September 2020)

#### Theme 8: Comfortable and willing to seek mental health help

A participant mentioned that the reason for not seeking mental health help was the type of help that would be received, as he assumed that it would be online via Zoom or an application. Participants (*n* = 4) felt that it would not be beneficial as working online is one of the factors causing mental health issues and an application may not be extensive enough to help. A participant reported: ‘If the university were to offer mental health services to students it would be online, via zoom and stuff and that would just be like working backwards’ (Participant 2, fourth-year student, 22 September 2020). Some mentioned that it would be better to sit down with a psychologist whom they are comfortable with and communicate directly. It was mentioned: ‘If it is physical, I think people would take (it) more seriously compared to online’ (Participant 2, fourth-year student, 22 September 2020). Others felt that they are more confident and will seek help via an application or social media. Many participants (*n* = 8) believed that peer support was very important as their peers are in a similar situation. Participants (*n* = 7) did mention that speaking about or seeking mental health help was not common. A participant reported: ‘Myself or my peers can be very scared or shy when communicating with others and speaking about mental health’ (Participant 3, second-year student, 25 September 2020). For example, when talking to some lecturers, they felt afraid and did not want to be judged or feel as if they were not smart enough. It was mentioned that helping their peers was beneficial as it had helped them view people from a new perspective and appreciate themselves and the people around them. When asked about using an application as a source of mental health help, a participant indicated: ‘That would actually be a good idea considering we don’t have much time physically to attend sessions’ (Participant 7, fourth-year student, 27 September 2020). Another participant mentioned:

‘I think that [*it*] would be good, it’s convenient, easily accessible. I think it’s helpful for people who have mild issues of mental health because I feel like for a person that has severe mental health issues you need to sit down with them and monitor their body languages and facial expressions’. (Participant 9, fourth-year student, 25 September 2020)

#### Theme 9: Factors that discourage or prevent students from seeking mental health help

Judgement and stigma were common factors mentioned by many participants that were hindrances to them seeking help. The lack of existing mental health services, transport and finances were other factors that were highlighted. It was also mentioned that those who seek mental health help may be seen as insane or mentally disturbed. Some cultures prefer not to foreground mental health issues and rather try to adapt to it. A participant mentioned:

‘I think families are very nonchalant about mental health issues, some people don’t even really believe it exist[*s*]. They believe you bring it on yourself, it’s not a mental illness’ (Participant 5, second-year student, 25 September 2020)

One participant indicated: ‘I think it comes a lot from people’s perception and understanding that mental health is an actual issue that a lot of people suffer from’ (Participant 2, fourth-year student, 22 September 2020). Another factor mentioned was the type of service provided when receiving mental health help as there are assumptions that some government service providers can be rude and ill-treat them. This resulted in individuals not wanting to return even if the government service provider does their job well. Some felt that they would not have time or feel too tired and overwhelmed to seek mental health help as it becomes an added stress. One participant reported: ‘The degree pushes you to such a limit like the edge of everything that you have to sacrifice your mental health just to get your degree’ (Participant 7, fourth-year student, 27 September 2020). The participant also mentioned: ‘We also have no time to attend any mental health consultations to help us because if you look at the timetable of all the years of optometry it’s full’ (Participant 7, fourth-year student, 27 September 2020). Participants also indicated that they may not want to speak about their mental health or trust the person they were seeking help from. One participant felt that the mental health help provided would depend on how much the student was willing to share and how willing they were to receive help. This was indicated as one participant mentioned:

[*S*]ometimes, you feel like you can handle it by yourself so there’s no need to tell someone else what’s happening, like how much you’re willing to share also how much you’re willing to receive help. (Participant 1, third-year student, 23 September 2020)

#### Theme 10: Returning to university and the possibility of sit-down exams

A participant reported: ‘Going back to university would be great and helpful’ (Participant 2, fourth-year student, 22 September 2020). Participants from rural areas indicated that they were not in an environment suitable for studying during the lockdown. Returning to university has motivated them to learn and revise more as well as being able to directly communicate with lectures and peers has allowed them to understand better and gain more practical skills. A participant reported:

‘Going back to university I am happy with that one, possibility of sit-down exams, I am also happy because I am able to write at my own pace and in a manner that is perfect and working for me’. (Participant 9, fourth-year student, 25 September 2020)

Most participants did prefer online tests or examinations as they felt that writing sit-down examinations would encourage them to revise more. Some participants (*n* = 3) reported that doing online tests at home made them feel depressed and alone compared to sit-down tests as they are seated alongside their peers. Some mentioned that the thought of sit-down examinations made them feel very anxious, stressed and overwhelmed. A few participants (*n* = 3) were concerned about returning to university and over the writing conditions of sit-down examinations because of COVID-19 pandemic whereas others were not as worried about returning as the COVID-19 infection rate was slowing down and the lockdown level was decreasing. This was indicated by one participant:

‘I’m not too concerned about going back now because the COVID-19 numbers have dropped, it’s not as bad, its level 1 so I don’t feel stressed about returning’. (Participant 5, second-year student, 25 September 2020)

## Discussion

According to the World Health Organization, mental health is related to mental and psychological well-being. It is more than the absence of a mental disorder and also includes the ability to think, learn and understand one’s emotions and the reactions of others.^11^ Mental health is a state of balance, both within and with the environment. The unpredictable and uncertain nature of the COVID-19 pandemic together with containment strategies including lockdowns, physical distancing regulations as well as the resulting economic breakdown could increase the risk of mental health problems and have psychological effects in individuals.^40,41^ These psychological effects can be expressed as depression, anxiety, stress, fear and worry amongst others.^40^ To this extent, several studies have investigated mental health in students,^24,31,42^ healthcare workers^32^ and the general population^30,33,34^ since the COVID-19 pandemic.

In this study, 97 participants (66%) had normal scores for the depression subscale and 50 participants (34%) had scores ranging from mild to extremely severe depression. These results are similar to a study involving Chinese inhabitants where it was reported that 70% of their sample (*n* = 1210) had a normal score for depression and of the remaining participants, 14% of them had scores indicating mild depression, 12% with scores indicating moderate depression and 4% had scores indicating severe to extremely severe depression.^33^ In a study^9^ conducted in Pakistan, 72% of participants (*n* = 61) showed a normal score for depression and only 7% had scores indicating severe to extremely severe depression and these findings compare favourably with the present study. Our results are in contrast to a study conducted in Wuhan that showed that 50.3% of participants (*n* = 191) reported significant symptoms of depression and included 33.0% with mild depression, 10.5% with moderate depression, 5.8% with moderately severe depression and 1.0% with severe depression.^43^ High levels of depression can have a negative impact on physical health. The relationship between depression and the perception of physical health was investigated recently and the findings endorse the addition of psychological treatment by medical practitioners for those who complain of infection-related symptoms in any outbreak.^44^

The current study found that 54% of participants had a normal anxiety score whilst 7% of them were found to have extremely severe anxiety. The remaining participants either had mild (9%), moderate (22%) or severe (8%) anxiety. The findings of our study are similar to a study in China involving university students where it was reported that 75% of them had normal anxiety scores and of the remaining participants, 1% experienced severe anxiety, 3% experienced moderate anxiety and 21% experienced mild anxiety when assessed using the Generalised Anxiety Disorder Scale questionnaire.^42^ Sundarasen et al.^24^ also reported similar results in their study involving Malaysian university students where most participants (92%) had a normal anxiety score, whilst 5% had scores indicating mild to moderate anxiety and 3% with scores indicating moderate to severe anxiety when assessed using the Zung self-rating anxiety scale questionnaire. This implies that the results of these two studies^24,42^ show the same trend as the present study wherein majority of participants comprising university students had normal anxiety scores despite using different questionnaires. Anxiety is the body’s natural response to a situation that causes stress and is a feeling of fear or apprehension about what is expected to follow. In our study, most of our participants presented with normal anxiety scores which suggest that they are responding normally to the COVID-19 pandemic and therefore would most likely be able to cope with the difficulties of the pandemic should the need arise. However, the 23 participants who had scores suggesting severe to extremely severe anxiety suggest that the impact of the pandemic is resulting in anxiety that is exceeding what the body can tolerate. This is worrying as increased anxiety can interrupt daily activities, affect both physical and psychological health and increase the risk of physical and mental health conditions.^45^

The majority of participants (73%) in this study had normal stress scores whilst 27% of participants had abnormal scores associated with mild (16%) moderate (7%), severe (3%) and extremely severe stress (1%). Wang et al.^33^ also reported that majority of their sample (68%) had normal stress scores and 32% had abnormal scores with mild stress scores being most common (24%). The similarity in stress scores between the present study and that by Wang et al.^33^ maybe observed as participants in both of these studies were not diagnosed with COVID-19 and consequently had relatively lower stress. In contrast, the findings of the present study are different to the results of a study conducted in Bangladesh in which it was found that only 40% of their participants had normal stress scores and 60% had abnormal scores associated with mild, moderate, severe and extremely severe stress.^30^ This discrepancy in results may be because of the smaller sample size in the current study (*n* = 147) compared with the Bangladesh study (*n* = 1427). Furthermore, the Bangladesh study included adult participants from the general population who may have been more concerned and stressed over job loss, economic activity and livelihood in addition to education. Stress is a natural, normal and necessary response when individuals face dangerous situations. In this study, most participants presented with normal stress scores and this suggests that they are responding to the COVID-19 pandemic in a way in which the internal drive of the human body is able to fight against the challenges of the COVID-19 pandemic. However, the five participants with scores suggesting severe to extremely severe stress may be experiencing an excessive adverse reaction and consequently may be limited in their ability to tolerate and cope with the consequences of the COVID-19 pandemic. This is worrying as stress of excessive magnitudes is likely to naturally disrupt the balance of physical and psychological functions and impair physical and mental health and even cause physical and mental illness.^41,46^

The DASS-21 questionnaire scores in relation to gender and level or year of study revealed interesting results. In the present study, male participants had higher mean scores for the depression, anxiety and stress subscales. However, most studies in the literature have reported the opposite trend that females have higher levels of depression, anxiety and stress.^9,24,30,33,34,43^ It has been suggested that differences in the processing of serotonin in the male and female brain may explain the gender differences observed for the symptoms of depression, anxiety and stress.^28^ In the present study, the sample consisted of majority females with a ratio of female to male participants of ~3.5:1 and this may account for the gender difference in findings between our study and other studies. With regard to the level or year of study, fourth year students had the highest mean scores on the depression (9.65), anxiety (9.00) and stress (12.25) subscales. As educational activities were hampered during the lockdown, it may be the reason for the higher levels of stress particularly amongst the fourth year students as this was the last year in their academic degree of study. In addition, factors such as uncertainty regarding academic progression, the possibility of not finishing the degree of study in planned time and uncertainty because of the consequences of COVID-19 on the economic sector in South Africa may also explain the higher levels of stress observed in fourth year students.^10^ Also, the fourth year students are comparatively older than the students in the other three years of study and therefore may have higher scores, particularly for stress, as it has been noted that stress scores increase with age.^47^

Even though participants reported that COVID-19 has affected their mental health in the interviews, the extent of its effect varied amongst the participants. There were many factors that contributed to the impact on mental health, such as online learning, concerns about academic performance, accommodation, finances and the effects of the lockdown, as has been highlighted by other college students.^48^ Participants felt that they were able to cope with their mental health without the help of external sources (peer support, counselling and psychologists). However, some participants reported that if the need arises, they would seek help from the relevant support services. Often many people may not seek help from external sources because of the stigma and judgement associated with mental health issues^48,49^ and this was also highlighted by some of the participants. Factors that deter participants from seeking mental health help from external sources include the lack of easily accessible and confidential assistance, finances, transport and the type of services (public or private services) that may be offered.^49^ In the interviews, some participants described the impact of COVID-19 on the completion of their degree of study as stressful and therefore had difficulties with concentration and this is likely to affect the participants confidence and increase stress and anxiety.^48^ The participants were also concerned about returning to university and having to learn many techniques in a short time period. This concern over increased workload was also reported in a recent study that collected interview data from college students in the United States of America.^48^ It was also observed that participants living in rural areas did not always have a suitable living environment to facilitate their online studies and this negatively impacted their educational activities. It is likely that the latter would have impacted the scores these participants obtained in the depression, anxiety and stress subscales. Some of the factors that could contribute to higher depression, anxiety and stress scores in students from rural areas include limited internet or network access, poor income, limited road access and the high cost of necessities in rural areas.^18^. In addition, many participants lived with their parents and particularly in rural areas. Consequently, these participants may have had extra responsibilities and stress associated with living with their parents as well as living in a rural environment.^18^

Most participants reported that the COVID-19 pandemic resulted in financial distress as family members had lost their jobs and the cost of accommodation was increasing. The combination of these two factors collectively resulted in higher amounts of stress for the participants and their families. According to participants, prolonged quarantine was also a factor that increased stress during this pandemic. Many participants were concerned about their loved ones and some would try to avoid them to prevent the spread of COVID-19. Some participants reported that a decrease in social interaction with their friends and families had an impact on their mental health as reported in recent studies.^42,48^ The majority of participants reported that they felt that their mental health was under control mostly because of strong support systems with friends and family. This is similar to the findings in a previous study in which one-third of participants indicated that communicating with their families and friends was the most common means used to cope with stress and anxiety during the COVID-19 pandemic.^48^

In South Africa, as the lockdown level progressed to level 1, participants reported high levels of stress, anxiety and fear related to returning to UKZN to examine patients as they would have to be in close contact with others. Roy et al.^10^ reported that ~40% of their respondents were affected by the thought of contracting the COVID-19 virus. In another study, the fear of being infected with COVID-19 was associated with anxiety as the number of suspected cases and people infected increased.^42^ Some participants reported that the use of face masks and face shields was uncomfortable and inconvenient for the examiner and the patient when in the clinical setting at UKZN. It was also observed that participants involved with activities like household chores and physical exercise reported that they coped better with the lockdown imposed by the current pandemic. This was also found in a previous study that showed the positive impact of physical activity on mental health and stress-coping capacity.^50^

Strengths of this study include the use of both quantitative and qualitative methods to collect data that were piloted and refined prior to data collection. Furthermore, all optometry students at UKZN were provided with data and consequently had access to the internet during the data collection phase. The latter contributed to eliminating any selection bias of only recruiting participants from a higher socio-economic background. The study also used the DASS-21 questionnaire that has been used in several other studies to assess mental health.^9,30,31,32,33^ Furthermore, the questionnaire was administered, and data were collected anonymously in an attempt to minimise the Hawthorne effect and any issues related to truthfulness of the responses. This study provides baseline data on the mental health, as assessed by depression, anxiety and stress subscales, of optometry students at a higher education institution. Limitations of the study include that participants were recruited using online convenience sampling as the questionnaire was administered using an online platform in adherence with the current social distancing regulations. Also, there were more females in the sample, probably as there are more female students registered in the optometry degree programme at UKZN, which limits the generalisability of the study results to other student populations with varying gender distributions. Furthermore, the study solely relied on self-reported responses, in relation to depression, anxiety and stress, regarding the participants’ experiences during the COVID-19 pandemic thus far and may not correspond with a true clinical diagnosis of a mental health professional.

## Conclusion

This study investigated the impact of COVID-19 on the mental health of optometry students at a higher education institution. The findings indicated that most participants had normal scores for depression, anxiety and stress; however, there were many that had abnormal scores for these three subscales as assessed with the DASS-21 questionnaire. Therefore, it is important to highlight the impact of the COVID-19 pandemic on mental health and subsequently provide mental health services, in the form of online support, student support groups and specialised college psychologists, to university students particularly males and those in higher levels of study. The results of this study should be used by higher education authorities to plan and implement appropriate strategies to help students to overcome the negative psychological consequences of the COVID-19 pandemic. This is important to ensure that optometry students safeguard their mental health, especially during this pandemic as this would directly affect their quality of life.
